# Combined Multivariate and Pathway Analyses Show That Allergen-Induced Gene Expression Changes in CD4^+^ T Cells Are Reversed by Glucocorticoids

**DOI:** 10.1371/journal.pone.0039016

**Published:** 2012-06-12

**Authors:** Yelin Zhao, Hui Wang, Mika Gustafsson, Antonella Muraro, Sören Bruhn, Mikael Benson

**Affiliations:** 1 Department of Clinical and Experimental Medicine, Faculty of Health Sciences, Linköping University, Linköping, Sweden; 2 Department of Pediatrics, University of Gothenburg, Gothenburg, Sweden; 3 Department of Pediatrics, Center for Food Allergy Diagnosis and Treatment, Veneto Region, University of Padua, Padua, Italy; University of Siena, Italy

## Abstract

**Background:**

Glucocorticoids (GCs) play a key role in the treatment of allergy. However, the genome-wide effects of GCs on gene expression in allergen-challenged CD4^+^ T cells have not been described. The aim of this study was to perform a genome-wide analysis to investigate whether allergen-induced gene expression changes in CD4^+^ T cells could be reversed by GCs.

**Methodology/Principal Findings:**

Gene expression microarray analysis was performed to profile gene expression in diluent- (**D**), allergen- (**A**), and allergen + hydrocortisone- (**T**) challenged CD4^+^ T cells from patients with seasonal allergic rhinitis. Principal component analysis (PCA) showed good separation of the three groups. To identify the correlation between changes in gene expression in allergen-challenged CD4^+^ T cells before and after GC treatment, we performed orthogonal partial least squares discriminant analysis (OPLS-DA) followed by Pearson correlation analysis. This revealed that allergen-induced genes were widely reversed by GC treatment (r = −0.77, P<0.0001). We extracted 547 genes reversed by GC treatment from OPLS-DA models based on their high contribution to the discrimination and found that those genes belonged to several different inflammatory pathways including *TNFR2 Signalling, Interferon Signalling, Glucocorticoid Receptor Signalling* and *T Helper Cell Differentiation*. The results were supported by gene expression microarray analyses of two independent materials.

**Conclusions/Significance:**

Allergen-induced gene expression changes in CD4^+^ T cells were reversed by treatment with glucocorticoids. The top allergen-induced genes that reversed by GC treatment belonged to several inflammatory pathways and genes of known or potential relevance for allergy.

## Introduction

Seasonal allergic rhinitis (SAR) is a common airway disease, which is caused by inhalant allergens such as birch or grass pollen. Following nasal exposure to the inhaled allergens, antigen presenting cells in the nasal mucosa present allergen peptides to CD4^+^ T cells [1]. In allergic patients, this results in activation and proliferation of allergen-specific T helper type 2 (Th2) cells. These cells release cytokines including IL-4, IL-5 and IL-13, which in turn activate B cells, eosinophils and mast cells [2], [3], [4], [5].

Glucocorticoids (GCs) are among the most effective drugs for controlling the inflammation in allergic rhinitis [6], [7]. Studies of nasal polyps and nasal fluids from patients with SAR show that GCs change the expression of hundreds of genes and proteins [8], [9]. In nasal mucosa, GCs exert anti-inflammatory effects by decreasing the number of Th2 cells and the production of Th2 cytokines, such as IL-4, IL-5 and IL-13. Interestingly, GCs also affect Th1 cells and the production of Th1 cytokines such as IL-2, IL-12 and IFN-γ [10], [11]. Moreover, GCs decreased the expression of IFN-γ and IFN-γ-induced genes in allergen-challenged CD4^+^ T cells from patients with SAR [12]. While GCs generally decrease the expression of inflammatory genes, GCs treatment may also increase the expression of anti-inflammatory genes [8]. However, the effects of GCs on gene expression in different cell types show considerable variation [13], [14], [15], [16], [17]. For example, GCs induce apoptosis in eosinophils, but have the opposite effect on neutrophils [18].

CD4^+^ T cells play crucial roles in allergic rhinitis. Allergen-challenged CD4^+^ T cells provide an *in vitro* model for studying the change in genes expression in CD4^+^ T cells in response to allergen challenge and to GC treatment [19]. Although several individual genes in CD4^+^ T cells from patients with SAR have been shown to be regulated by allergen challenge and GC treatment [12], the genome-wide effects of GCs on the changes in genes expression in CD4^+^ T cells with allergen challenge have, to our knowledge, not yet been described. In this study, we hypothesized that GCs reverse gene expression changes in allergen-challenged CD4^+^ T cells. To test this hypothesis, we examined gene expression microarray data from CD4^+^ T cells from patients with SAR. The cells were challenged with either diluent (D), allergen (A) or allergen + hydrocortisone (T) [12]. The data was analyzed with multivariate- and pathway analysis [9].

## Materials and Methods

### Ethics statement

This research has been approved by the regional ethics committee of the University of Gothenburg and the ethics committee of the University of Padua. We obtained written informed consent from all subjects. The written consent was obtained on a special form with information about the study and the conditions of the study, according to instructions from the ethics committee (this form was also approved by the ethics committee, which considers this of great importance for their approval).

### Subjects

First, samples from twelve patients with SAR outside the pollen season were analyzed and will be referred to as the training set. The two independent test data sets consisted of 21 patients and 28 patients with SAR outside the pollen season (these materials will henceforth be referred to as Test1 and Test2, respectively). The median (range) age of the training set was 23(20–25) and 3 were women. For the Test1, the median (range) age was 27 (16–46) and 12 were women. For the Test2, the median (range) age was 32 (15–47) and 10 were women. Exclusion criteria included a history of perennial symptoms and treatment with local or systemic corticosteroids or antihistamines for the last 2 months. The training set and Test2 were recruited from Sahlgrenska University hospital in Göteborg, Sweden while the Test1 cohorts were collected at Padua University Hospital, Padua, Italy.

### Allergen challenge

In the training set, peripheral blood mononuclear cells (PBMCs) obtained from 12 patients were challenged with diluent (D), allergen extracts from grass pollen (ALK Abello', Hørsholm, Denmark; 100 ug/mL) (A), or allergen + hydrocortisone (10^−7^ M, Sigma-Aldrich, St. Louis, Missouri, USA) (T) for 7 days in RPMI 1640 supplemented with 2 mM L-glutamine (PAA Laboratories, Linz, Austria), 5% human AB serum (Lonza, Switzerland), 5 µM β–mercaptoethanol (Sigma-Aldrich, St. Louis, Missouri, USA) and 50 ug/mL gentamicin (Sigma-Aldrich, St. Louis, Missouri, USA). In the Test1, PBMCs obtained from 21 allergic patients outside the pollen season were challenged with diluent (Test1 D) or allergen extracts from grass pollen (Test1 A) for one week. In the Test2, PBMCs obtained from 28 allergic patients outside the pollen season were challenged by allergen extracts from birch (one patient) or grass pollen for one week with (Test2 T) or without (Test2 A) GC treatment.

### Gene expression microarray analysis

CD4^+^ T cells were isolated using the CD4 Microbeads (Miltenyi Biotec, Bergisch-Gladbach, Germany) according to the manufacturer's protocol. The typical purity of sorted CD4^+^ T cells was >90%. Total RNA was extracted using the miRNeasy mini kit (QIAGEN, Valencia, CA, USA) according to manufacturer's instructions. The quantity of RNA was measured with a NanoDrop ND-1000 UV Spectrophotometer (NanoDrop Technologies, Wilmington, DE, USA). The RNA quality was examined in an Agilent 2100 Bioanalyzer using the RNA 6000 Pico kit and the RNA Integrity Number was calculated in Agilent 2100 Bioanalyzer expert software (Agilent Technologies, Palo Alto, CA, USA). Gene expression microarrays (Illumina, San Diego, CA, USA) were performed as previously described for the training and test cohorts [8], [20].

### Data analysis

Principal Component Analysis (PCA) was used to examine the systematic trends within the GEM data. Orthogonal partial least squares discriminant analysis (OPLS-DA) was performed to identify the correlation between changes in gene expression in allergen-challenged CD4^+^ T cells with or without GC treatment (see detailed methods in **Method S1**). The SUS-plot that combines the Cor(T_p_, X) profiles from two models where classes were compared to a common reference (group **A**) was used to identify the shared and unique structure between classes (**Figure S1**). The shared genes between two models with a |Cor(T_p_, X)|≥0.5 were extracted for pathway analysis. Both PCA and OPLS-DA were performed with SIMCA-P+12.0.1 software (UMETRICS, Umeå, Sweden). Hierarchical clustering analysis was performed with GeneSpring GX software (Agilent Technologies, Palo Alto, CA, USA). Ingenuity Pathways Analysis (IPA) software (http://www.ingenuity.com/) was used to map genes onto known canonical and cellular and molecular pathways [20]. We employed a Fisher's exact test to calculate a P value determining the probability whether the association between the proteins in the dataset and the canonical pathway can be explained by chance alone. Pathways with a P value less than 0.05 were considered to be statistically significant. Pearson correlation analysis was performed using the Statistical Package for the Social Sciences (SPSS) statistical software for Windows, Version 19.0.

## Results

### Outline of the study

Briefly, we first analyzed a training set consisting of diluent-, allergen- or allergen + GC-challenged CD4^+^ T cells from the allergic patients with gene expression microarrays (GEM). The aims were to determine if a) genes whose expression was changed by allergen would be reversed by treatment and b) which pathways those genes belonged to. Finally, we showed that the observed gene expression changes were supported by analysis of two independent materials, one of which consisted of allergen- or diluent- challenged CD4^+^ T cells (Test1) and another consisting of allergen and allergen + GC-treated CD4^+^ T cells (Test2).

### Principal Component Analysis to obtain an overview of the effects of allergen and glucocorticoids on gene expression in CD4+ T cells from patients with seasonal allergic rhinitis

**Figure 1:**
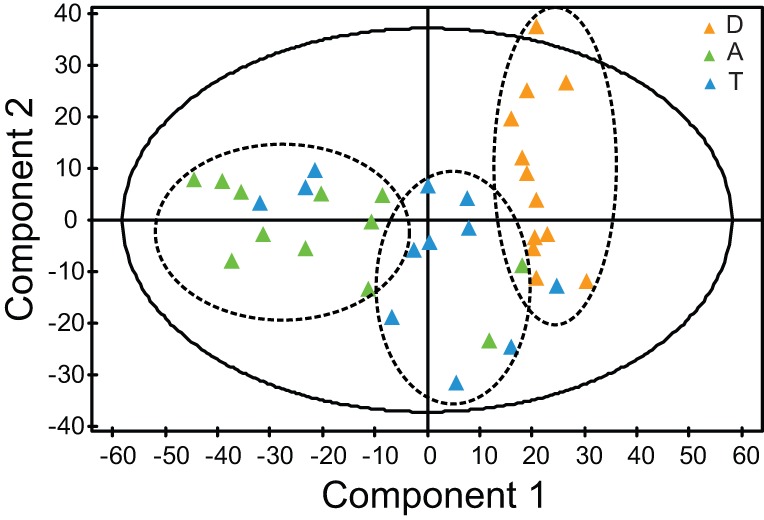
PCA modeling of the gene expression microarray data from diluent- (D), allergen- (A) or allergen + GC treated (T) CD4^+^ T cells from patients with seasonal allergic rhinitis in the training set.

PBMCs from the training set, namely 12 asymptomatic allergic patients out of pollen season were challenged with diluent (D), allergen (A) or allergen + hydrocortisone (T). CD4^+^ T cells extracted from the PBMCs were analyzed with GEM. PCA of the GEM data was performed to obtain an overview of the effects of the three conditions on gene expression. The PCA resulted in a model with 4 cross-validatory components. The total explained X variation (the gene expression data) was 53% and the predicted X variation was 36%. The PCA scatter plot with the first two components showed that the three groups (D, A and T) formed three distinct clusters. Allergen and diluent challenged cells were most separated, while the GC treated group, T, was located between D and A (**Figure 1**). This indicated that GCs tended to reverse allergen-induced gene expression changes in CD4^+^ T cells from allergic patients.

### Identification of allergen-induced gene expression changes that were reversed by GC treatment

**Figure 2:**
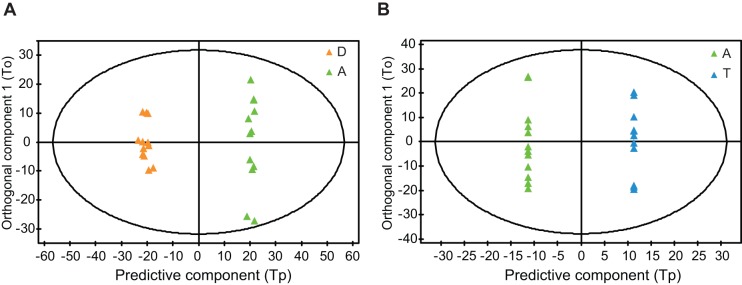
OPLS-DA modeling of diluent- (D), allergen- (A) or allergen + GC treated (T) CD4^+^ T cells from patients with seasonal allergic rhinitis in the training set. **A**) OPLS of D and A (Model DvsA); **B**) OPLS of A and T (Model AvsT).

We first performed OPLS-DA to identify the genes that most separated allergen or diluent challenged groups, A and D. Next, we repeated the analysis to identify the corresponding genes in allergen and allergen + GC challenged cells, A and T. OPLS-DA identifies correlation patterns that discriminate groups and estimates the relative importance of each included gene expression value for the discrimination [21], [22], [23]. OPLS-DA of D and A provided an OPLS model with one predictive component and three orthogonal components (Model DvsA) (**Figure 2A**). This model was significant based on ANOVA of the cross-validated residuals (CV-ANOVA) (P<0.001) [24]. The total explained Y variation was 99.6% and the total predicted Y variation was 81.6%. Of the total explained X variation of 52.8%, 24.7% predicted the discrimination between the groups D and A.

OPLS-DA of A and T provided an OPLS model with one predictive component and seven orthogonal components (Model AvsT) (**Figure 2B**). The CV-ANOVA showed that the Model AvsT was significant (P<0.001). The total explained Y variation was 100% and the total predicted Y variation was 42.2%. Of the total explained X variation of 64.9%, 8.4% of the genes predicted the discrimination between A and T.

**Figure 3:**
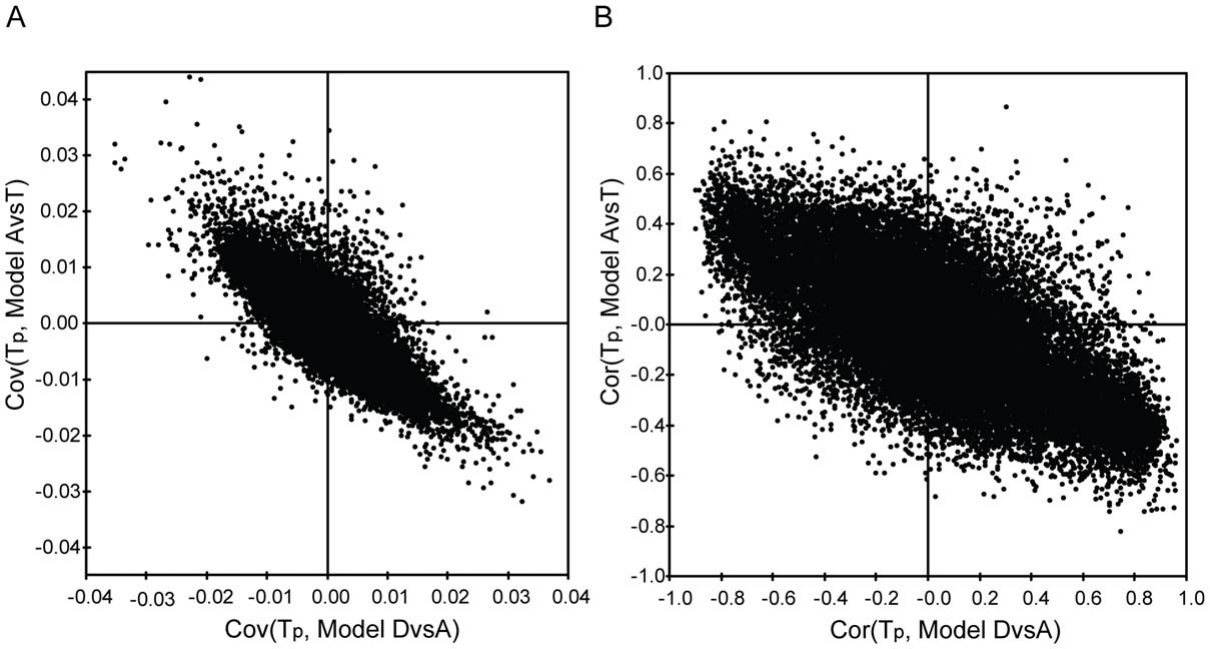
Comparison between Model DvsA and Model AvsT derived from the training set. **A**) Comparison of the Cov(Tp) of all genes between Model DvsA and Model AvsT; **B**) Comparison of the Cor(Tp) of all genes between Model DvsA and Model AvsT. D, diluent-challenged CD4^+^ T cells; A, allergen-challenged CD4^+^ T cells; T, allergen-challenged + GC treated CD4^+^ T cells. Cov(Tp), the covariance of the predictive component; Cor(Tp), the correlation of the predictive component.

In order to understand how GC treatment affected allergen-induced gene expression in CD4^+^ T cells, we analyzed the correlation between the covariance of all genes from the two models by Pearson correlation analysis. The data illustrated an inverse correlation (r = −0.77, P<0.0001) (**Figure 3A**). In order to extract the genes that were most reversed by GCs, we used a SUS-plot, which combined the Cor(T_p_) value of each gene from the two models (Model DvsA and Model AvsT). The SUS-plot also showed an inversely shared gene structure between the two models (r = −0.97, P<0.0001) (**Figure 3B** **, Table S1**). We extracted 547 genes that were found to be most reversed by GCs based on a |Cor(T_p_)| ≥0.5 (**Figure 3B** **, Table S2**) [25].

### Pathway analysis of allergen-induced genes that were most reversed by glucocorticoids

**Figure 4:**
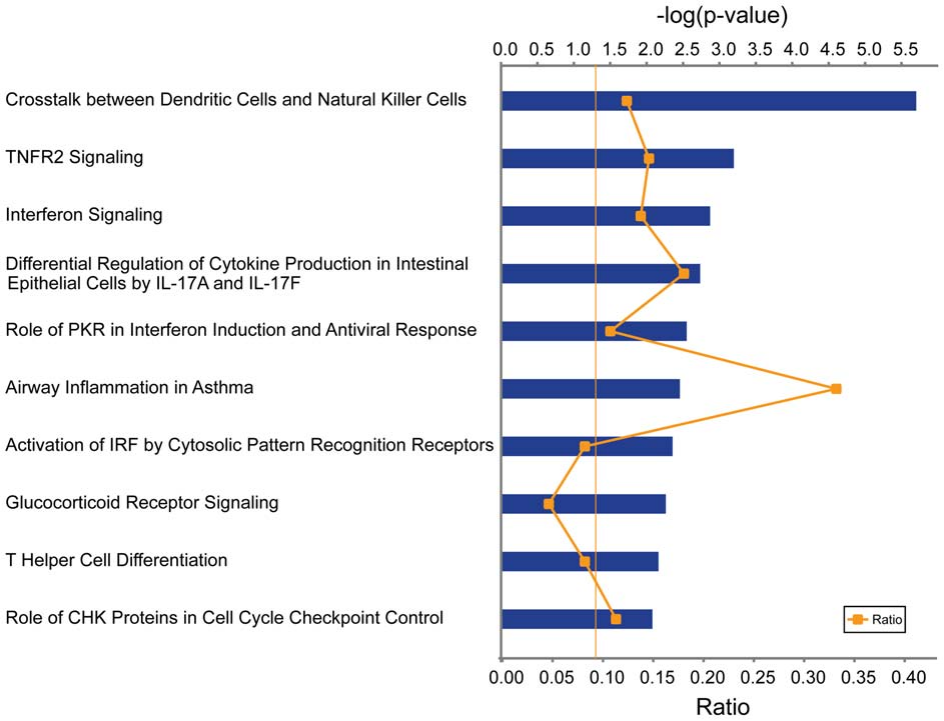
Pathway analysis of allergen-induced top 547 genes whose expression was reversed by glucocorticoids. The top 547 genes with a |Cor(Tp)|≥0.5 from the two models were extracted and mapped to Ingenuity pathway analysis. The yellow threshold indicates 95% confidence.

We performed pathway analysis of the 547 allergen-induced genes whose expression was reversed by GC treatment, using the IPA software. The top pathways enriched for these genes included *TNFR2 Signalling, Interferon Signalling, Differential Regulation of Cytokine Production in Intestinal Epithelial Cells by IL-17A and IL-17F, Airway Inflammation in Asthma, Glucocorticoid Receptor Signalling* and *T Helper Cell Differentiation* (**Figure 4**). We also investigated the cellular and molecular function of these genes using IPA. The top five cellular and molecular pathways included *cell death, cellular growth and proliferation, cellular development, gene expression and cell-to-cell signalling and interaction* (**Table 1**). These pathways included inflammatory genes of known importance for allergy, such as *CSF2, TNF*, *IFNG, GZMA, GZMB, IRF4, STAT1 and IL13*, or potential relevance for allergy such as *NR3C1* and *IL21R* (**Table 2**). Taken together, these findings indicated that GC treatment reversed gene expression changes in a wide variety of pathways and genes in allergen-challenged CD4^+^ T cells.

**Table 1:** Cellular and molecular functions of top 547 genes whose expression changed by allergen and reversed by GCs.

Name	P value	Molecules
Cell Death	2.6E-10–2.0E-03	122
Cellular Growth and Proliferation	4.3E-07–1.9E-03	147
Cellular Development	1.3E-06–2.0E-03	101
Gene Expression	3.3E-06–2.1E-03	111
Cell-To-Cell Signaling and Interaction	3.5E-06–1.8E-03	67

**Table 2:** Genes of known relevance for allergy, whose expression increased following allergen-challenge and were reversed by treatment with glucocorticoids.

Gene symbols	Model DvsA		Model AvsT	
	Cov(Tp)	Cor(Tp)	Cov(Tp)	Cor(Tp)
*CSF2*	0.034	0.95	−0.027	−0.54
*TNF*	0.018	0.95	−0.017	−0.63
*IFNG*	0.022	0.88	−0.022	−0.55
*GZMA*	0.023	0.81	−0.028	−0.56
*GZMB*	0.030	0.89	−0.025	−0.50
*IRF4*	0.025	0.92	−0.022	−0.52
*STAT1*	0.022	0.85	−0.025	−0.71
*IL13*	0.013	0.87	−0.018	−0.69
*NR3C1*	0.009	0.66	−0.011	−0.53
*IL21R*	0.016	0.79	−0.021	−0.60

Cov(Tp), the covariance of the predictive component; Cor(Tp), the correlation of the predictive component.

### Analyses of allergen-induced gene expression changes that were reversed by treatment with glucocorticoids in independent materials

**Figure 5:**
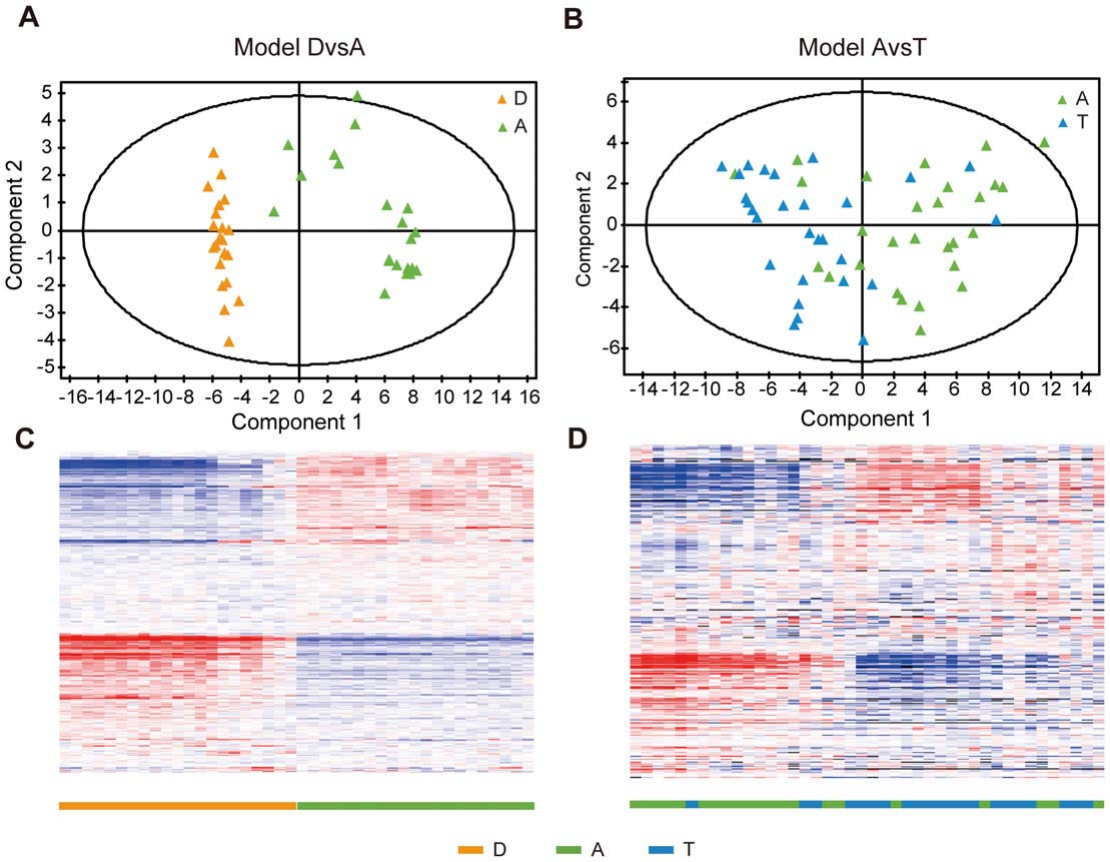
Validation studies of top 547 genes whose expression changed in CD4^+^ T cells from allergic patients after allergen-challenge and were reversed by treatment with glucocorticoids. The CD4^+^ T cells from allergic patients were obtained from two independent materials and analysed with gene expression microarrays. PCA (**A** and **B**) and hierarchical clustering analysis (**C** and **D**) of Test1 (**A** and **C**) and Test2 (**B** and **D**) with the top 547 genes that were changed by allergen challenge and were reversed by GC treatment.

The observed gene expression changes were supported by analyses of two independent materials, one material consisted of diluent- and allergen-challenged (D and A) CD4^+^ T cells from 21 allergic patients (Test1) while the other one consisted of CD4^+^ T cells challenged with allergen (A), or allergen + GC (T) from 28 allergic patients (Test2). Because of the large number of genes (n = 547) the studies were performed with gene expression microarrays. PCA and hierarchical clustering showed that the 547 genes clearly separated the two groups (**Figure 5**).

We also performed OPLS-DA with the gene expression microarray data from the two Test sets followed by Pearson correlation analysis of the covariance of all the genes in the two OPLS-DA models. These analyses also showed an inverse correlation (r = −0.48, P<0.0001), in consistency with the analyses of the initial material (**Figure S2**).

## Discussion

GCs have an important role in the treatment of allergy and other inflammatory disorders. GCs have wide-ranging effects on different tissues and cell types [26], [27], [28]. Interestingly, while GCs mainly decrease the expression of inflammatory genes, they may also increase the expression of anti-inflammatory genes. It is also of note that the expression of several inflammatory genes is not affected [8]. From a clinical perspective, 10–30 % of patients with different inflammatory diseases do not respond to GC treatment [27], [29]. This suggests the need to determine the genome-wide effects of GCs on disease-related genes as well as which pathways those genes belong to. Despite the importance of GCs in treating allergy, there is limited information on the genome-wide effects of GCs in cells and tissues from allergic patients. One recent study of nasal fluids, fluid cells and mucosa showed large and diverse effects on the mRNA and protein expression levels [20]. A previous analysis of nasal polyps, which have immunohistochemical similarities with allergic inflammation, also showed large effects of GCs that were mainly inhibitory, but also in some cases enhancing [8].

CD4^+^ T cells from patients with SAR provide an *in vitro* model for studying genes expression changes in response to allergen challenge and how these are affected by GCs. In this study, we examined this model with gene expression microarray-, multivariate and pathway analysis. The reason for using multivariate analysis was that univariate analysis does not take into account the relationships between variables and their correlations to the classification between groups. Compared to univariate analysis, multivariate analysis allows us to interpret and visualize multiple variables, such as gene expression microarray data, providing integrated information with less error and more validity. For instance, PCA modeling of the GEM data enabled us to find that the gene expression pattern induced by allergen challenge was potentially reversed by GC treatment. Using the OPLS-DA, we further indentified the covariance and correlation of the whole genes to the classification caused by allergen challenge and GC treatment. The scatter plot of the covariance of the whole genes and also the SUS plot allowed us to identify that the expression pattern of whole genes in allergen-challenged CD4^+^ T cells was systematically reversed by GC treatment. These plots also permitted us to determine allergen-induced genes that were reversed by GC treatment.

We found that, in general, allergen-induced genes expression changes were reversed by GC treatment. The genes that were most affected by allergen- and reversed by GCs belonged to a wide variety of inflammatory pathways and cellular functions. Several of those pathways have known roles in allergy, namely *TNFR2 Signalling, Interferon Signalling, Differential Regulation of Cytokine Production in Intestinal Epithelial Cells by IL-17A and IL-17F, Airway Inflammation in Asthma, Glucocorticoid Receptor Signalling* and *T Helper Cell Differentiation*. Previous studies have shown important roles for several genes in those pathways. For example, *IFNG* is a master cytokines of Th1 differentiation, while *IRF4* has a key role in Th2 differentiation [30], [31]. The soluble IL-2RB is known to reflect T cell involvement and was found to be increased in patients with allergic disease but to be reduced by GC treatment [32]. Our approach may also help to identify novel candidate genes in allergy. The rationale for this assertion is that a gene, whose expression is induced by allergen and also reversed by GCs, has an increased likelihood to be relevant for the disease. Examples of such genes included nuclear receptor subfamily 3, group C, (*NR3C1*), which is also known as the glucocorticoid receptor, from the *Glucocorticoid Receptor Signalling*, which can be activated by glucocorticoids [33]. It has recently been reported that NR2C1 increased in nasal mucosal from patients with allergic rhinitis [34]. IL-21 receptor (IL-21R) from the *T helper cell differentiation pathway* is important in the development of Th2 response and has been demonstrated to be essential for allergic skin inflammation in humans and mice [35], [36]. Similar to our findings, a previous study gene expression microarray study of CD4+ T cells from patients allergic to house dust mite and healthy showed a Th2-skewed gene expression profile in the patients [37].

Limitations of our study include that a large number of genes are analyzed in a small number of patients. This involves the risk of spurious findings. This suggests the need for validation studies. However, the large number of genes affected by allergen and GCs makes validation with low throughput techniques like QPCR impractical. Other possible confounding factors could be that cohorts from two different countries were examined, and that young men were over-represented in the training set. To our knowledge the influences of ethnic and racial differences on the expression of genes relevant for allergy have not been characterized. Such differences could have impact on treatment response. Future studies are therefore warranted to elucidate such influences. Regarding the effects of gender, previous studies by us and others have shown that gender may influence gene- and protein expression changes [38]. In this study, we tested the relevance of our findings in two independent materials, one which consisted of allergen- and diluent challenged CD4^+^ T cells and another consisting of allergen- and GC treated CD4^+^ T cells. These analyses showed highly significant correlations with the results from the first study and thereby supported the general relevance of our findings. A technical limitation of the present study is that we used positive sorting of CD4^+^ T cells by magnetic-activated cell sorting (MACS), which results in slightly lower purity than positively sorted cells by fluorescence-activated cell sorting (FACS).

In conclusion, allergen-induced gene expression changes in CD4^+^ T cells were reversed by treatment with glucocorticoids. The top allergen-induced genes that reversed by GC treatment belonged to several inflammatory pathways and genes of known or potential relevance for allergy.

## Supporting Information

Figure S1 **Illustration of the SUS-plot.** A SUS-plot combining the Cor(**T**_p_, X) from OPLS-DA model 1 and 2 was illustrated. X variables in the diagonal **A** were shared by both models while X variables in the diagonal **B** were inversely shared. X variables in the square **C** and **D** were unique in the model 1 and 2, respectively.
(EPS)

Figure S2 **Inversely shared gene expression structure between the two independent materials.** The scatter plot combines the Cov (**T**_p_) of all genes in Model DvsA from the Test1 and Model AvsT from the Test2.
(TIF)

Methods S1 **Orthogonal partial least squares discriminant analysis.**
(DOC)

Table S1 **All genes analyzed in OPLS-DA.**
(XLS)

Table S2 **Inversely correlated genes with high reliability.**
(XLS)
