# Supplementary figures and images for: Combined Multivariate and Pathway Analyses Show That Allergen-Induced Gene Expression Changes in CD4+ T Cells Are Reversed by Glucocorticoids
Source: PLoS One. 2012 Jun 12;7(6):e39016. doi: 10.1371/journal.pone.0039016 (PMC3373548; doi:10.1371/journal.pone.0039016)

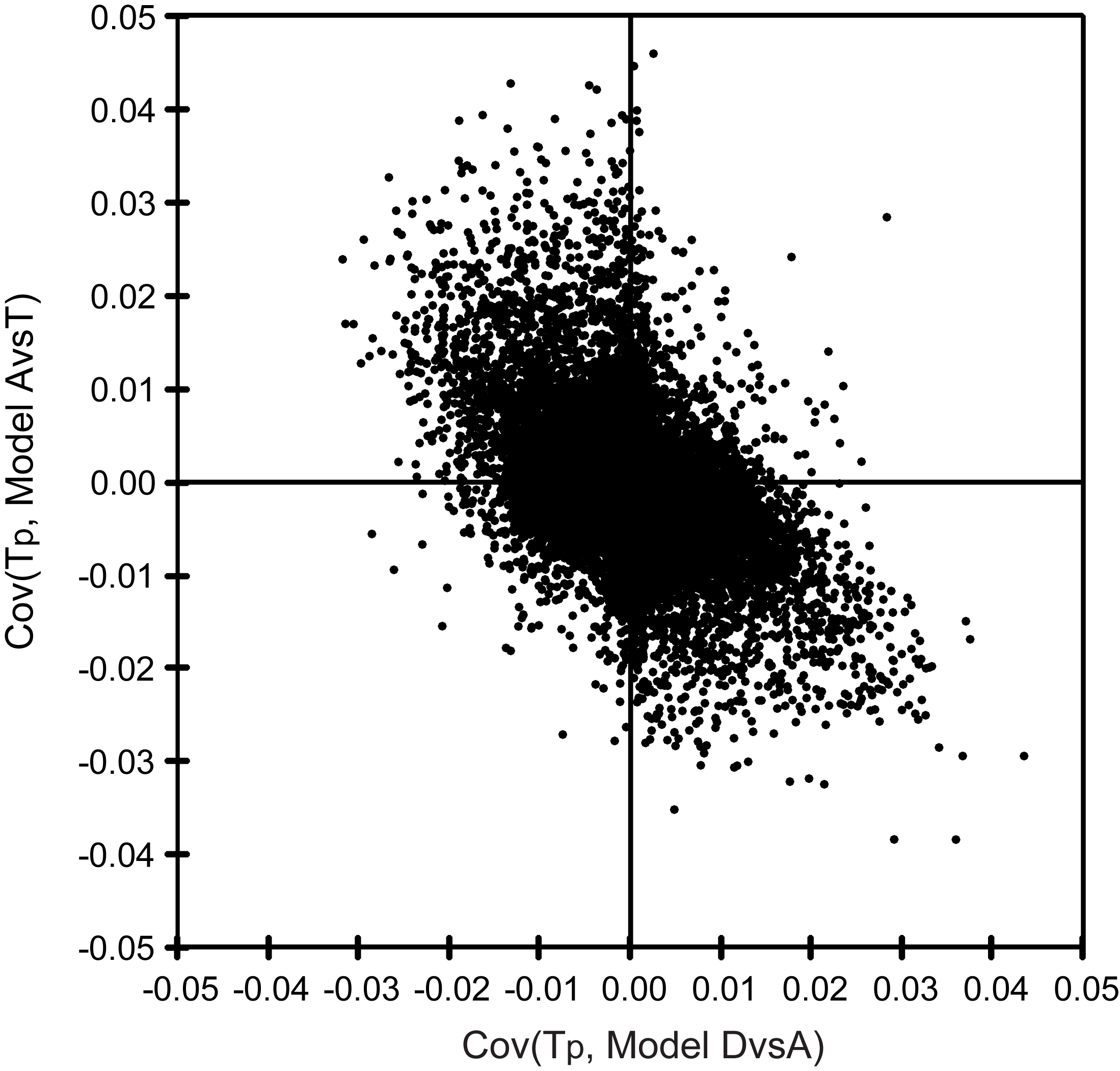

Supplement: Figure S2 — Inversely shared gene expression structure between the two independent materials. The scatter plot combines the Cov (T p) of all genes in Model DvsA from the Test1 and Model AvsT from the Test2. (TIF) [file pone.0039016.s002.tif]
