# Supplementary material for: Combined Multivariate and Pathway Analyses Show That Allergen-Induced Gene Expression Changes in CD4+ T Cells Are Reversed by Glucocorticoids
Source: PLoS One. 2012 Jun 12;7(6):e39016. doi: 10.1371/journal.pone.0039016 (PMC3373548; doi:10.1371/journal.pone.0039016)
Supplement: Methods S1 — Orthogonal partial least squares discriminant analysis. (DOC) [file pone.0039016.s003.doc]

**Orthogonal partial least squares discriminant analysis**

Orthogonal partial least squares discriminant analysis (OPLS-DA) is a supervised multiple regression analysis for identification of discrimination between different datasets referred to as X (here the gene expression microarray data) and Y (here a ternary vector with the value 0 for the group **D**, 1 for the group **A** and 2 for the group **T)**. OPLS-DA dissects the systematic X variation into two parts, plus the residual noise, one that is correlated to the Y and the other one that is un-correlated to the Y . Hence, the OPLS-DA model comprises of two blocks of modeled variation: the Y predictive component (**T**_p_**P**_p_), that represents the between class variation and the Y orthogonal component (**T**_o_**P**_o_), representing the within class variation. In our study **T** represents the score matrix and **P** represents the loading matrix. The OPLS-DA model was estimated by a leave out data Cross-Validation (all data were left out once in a 7 leave out series and only once). Prior to modeling, the GEM data were pre-processed with log-transformation and Pareto scaling, the latter allows for reducing the impact of noise and artefacts in the model . The S-plot of the predictive component of the OPLS-DA model combines the contribution (covariance, Cov(**T**_p_, X)) and the reliability (correlation, Cor(**T**_p_, X)) in one score plot for interpretation of the predictive component. The SUS-plot that combines the Cor(**T**_p_, X) profiles from two models where classes were compared to a common reference (group **A**) was used to identify the shared and unique structure between classes. An illustration of the SUS-plot was demonstrated (**Figure S1**). Genes close to the diagonal were shared between classes and genes outside the diagonal were unique for the specified class. Genes in the diagonal **A** (**Figure S1**) were up or down in both classes while genes in the diagonal **B** (**Figure S1**) were opposite in both classes.

**References**

1. Whelehan OP, Earll ME, Johansson E, Toft M, Eriksson L (2006) Detection of ovarian cancer using chemometric analysis of proteomic profiles. Chemometrics and intelligent laboratory systems 84: 82-87.

2. Vinay P, Allignet E, Pichette C, Watford M, Lemieux G, et al. (1980) Changes in renal metabolite profile and ammoniagenesis during acute and chronic metabolic acidosis in dog and rat. Kidney int 17: 312-325.

3. Wiklund S, Johansson E, Sjostrom L, Mellerowicz EJ, Edlund U, et al. (2008) Visualization of GC/TOF-MS-based metabolomics data for identification of biochemically interesting compounds using OPLS class models. Anal Chem 80: 115-122.
